# Changes in risk factors for non-communicable diseases associated with the ‘Healthy choices at work’ programme, South Africa

**DOI:** 10.1080/16549716.2020.1827363

**Published:** 2020-10-20

**Authors:** Darcelle Schouw, Robert Mash, Tracy Kolbe-Alexander

**Affiliations:** a Division of Family Medicine and Primary Care, Faculty of Medicine and Health Sciences, Stellenbosch University, Cape Town, South Africa; b Sport and Exercise Science, School of Health and Wellbeing, University of Southern Queensland, Ipswich, Queensland, Australia; c School of Human Movement and Nutrition Sciences, The University of Queensland, Brisbane, Australia; d Division of Exercise Science and Sports Medicine, Department of Human Biology, Faculty of Health Sciences, University of Cape Town, Cape Town, South Africa

**Keywords:** Non-communicable diseases, workplace health promotion, risky behaviour, risk factors, cardiovascular disease

## Abstract

**Background:**

Globally 71% of deaths are attributed to non-communicable diseases (NCD). The workplace is an opportune setting for health promotion programs and interventions that aim to prevent NCDs. However, much of the current evidence is from high-income countries.

**Objective:**

The aim of this study was to evaluate changes in NCD risk factors, associated with the Healthy Choices at Work programme (HCWP), at a commercial power plant in South Africa.

**Methods:**

This was a before-and-after study in a randomly selected sample of 156 employees at baseline and 137 employees at 2-years. The HCWP focused on food services, physical activity, health and wellness services and managerial support. Participants completed questionnaires on tobacco smoking, harmful alcohol use, fruit and vegetable intake, physical activity, psychosocial stress and history of NCDs. Clinical measures included blood pressure, total cholesterol, random blood glucose, body mass index, waist circumference and waist-to-hip ratio. The 10-year cardiovascular risk was calculated using a validated algorithm. Sample size calculations evaluated the power of the sample to detect meaningful changes in risk factors

**Results:**

Paired data was obtained for 137 employees, the mean age was 42.7 years (SD 9.7) and 64% were male. The prevalence of sufficient fruit and vegetable intake increased from 27% to 64% (p < 0.001), those meeting physical activity guidelines increased from 44% to 65% (p < 0.001). Harmful alcohol use decreased from 21% to 5% (p = 0.001). There were clinical and statistically significant improvements in systolic and diastolic blood pressure (mean difference −10.2 mmHg (95%CI: −7.3 to −13.2); and −3.9 mmHg (95%CI: −1.8 to −5.8); p < 0.001) and total cholesterol (mean difference −0.45 mmol/l (−0.3 to −0.6)). There were no significant improvements in BMI. Psychosocial stress from relationships with colleagues, personal finances, and personal health improved significantly. The cardiovascular risk score decreased by 4.5% (> 0.05).

**Conclusion:**

The HCWP was associated with clinically significant reductions in behavioural, metabolic and psychosocial risk factors for NCDs.

## Background

Globally 71% of deaths are attributed to non-communicable diseases (NCDs), with over 85% of premature deaths occurring in low-and middle-income countries [1,2]. Premature deaths from NCDs in people of working age are expected to increase by 41% in developing economies between 2000 and 2030 [3]. These diseases accounted for 51% of all deaths in South Africa in 2016 [4].

The increased prevalence in NCDs is associated with unhealthy lifestyles, an ageing workforce and changing socio-economic determinants [3,5–7]. NCDs are ascribable to four behavioural risk factors: excessive alcohol use, tobacco smoking, unhealthy diet and inadequate physical activity [8]. Behavioural, metabolic (blood pressure, cholesterol, diabetes, body mass index, waist circumference and waist-to-hip ratio) and psychosocial risk factors (causes of stress at home and in the workplace) are associated with NCDs such as cardiovascular disease, cancers, diabetes and chronic lung disorders [8].

The World Health Organization’s (WHO) Global Plan of Action on Workers Health, highlights the importance of the workplace for the prevention of NCDs [8,9]. A substantial proportion of the adult population can be reached in the workplace [10]. The workplace environment shapes employees’ health and safety, influences their health behaviours through physical and psycho-social mechanisms and can influence the risk of developing NCDs [9]. Employees’ socio-economic status, the impact of work stressors and their personal health risk profile influences their risk for NCDs [11].

The WHO recommends a number of interventions in the workplace such as reducing exposure to second-hand tobacco, providing low sodium food options and education on increased fruit and vegetable intake as well as multicomponent physical activity programmes [12]. The workplace should enable employees to make the healthy choice, the easy choice [13]

Behavioural risk factors for NCDs are highly prevalent amongst the South African population, including those that are employed [14,15]. Occupational psychosocial stress factors such as high workload, psychological demands, loss of autonomy, poor recognition, lack of social support and rewards, are all associated with employee heath and psychological wellbeing [16]. Mental and emotional stress contributes to NCDs with occupational stress identified as a global workplace challenge [17]. Changing the psychosocial work environment can contribute to enhanced wellbeing, work-life balance and job security [18].

Workplace health promotion programmes (WHPP) have been effective in reducing absenteeism, improving productivity, and reducing behavioural, metabolic and psychosocial risk factors for NCDs [9,19]. WHPP also reduce co-morbidity in people with existing NCDs [20]. Most WHPP focus on conducting health risk assessments in office-based settings and among self-selected participants in a traditional health service model [21]. More evidence is needed of the benefits of transforming the organisational environment as a whole with new methodologies and approaches [15,21,22]Despite evidence on the benefits of WHPPs only 34% of African countries have a clear inter-sectoral policy for addressing risk factors and NCDs in the workplace [12].

Further research and policy development is required to transform the workplace-based business, physical and psychosocial environments to reduce NCDs [23]. Priority should be given to improving WHPP infrastructure, employee participation in WHPPs and research on WHPPs to establish best practice standards [13]. The aim of this study was to evaluate changes in NCD risk factors associated with the introduction of a WHPP in a South African workplace.

## Methods

### Study design

This was a before-and-after study that evaluated changes in risk factors for NCDs, associated with the introduction of the Healthy Choices at Work programme (HCWP) at a commercial power plant in the Western Cape, South Africa. A baseline survey showed that employees at the power plant had multiple risk factors for NCDs and a third of the workforce had a moderate-high risk of death from stroke or myocardial infarction in the next 10-years [15]. Participatory action research enabled the design, development and implementation of the HCWP [24].

### The setting

This commercial power plant, situated close to the City of Cape Town in a nature reserve, had 1743 permanent employees. The power plant operated in a highly pressurised environment due to the lack of generation capacity in South Africa which was relative to the increased demand for electricity, and the dissatisfaction of the population with regular load shedding. Employees had a wide range of occupations, which included engineers, plant operators, physicists, technicians, artisans (tradesperson), support staff and shift workers. The management team consisted of an executive committee with 10–12 senior managers led by a general manager. The power plant’s Health and Wellness department provided occupational health services and conducted annual health risk assessments on self-selected employees. Food was subsidised and provided by an external company who were contracted to run the canteen and vending machines.

### Study population and sample size calculation

The study population included all permanent employees working at the power plant and there were no exclusion criteria. A representative sample of 156 employees had been included in a prior survey to evaluate the prevalence of NCD risk factors in the organisation [15]. This same sample was included in this before-and-after study to evaluate the effect of the HCWP. Sample size calculations were performed to evaluate the power of this sample to detect meaningful changes in risk factors for NCDs over time. Assuming an alpha error of 5%, a sample size of 156 gave 98% power to detect a mean 5 mmHg decrease in diastolic blood pressure, 95% power to detect a reduction from 50% to 30% prevalence of physically inactive employees (threshold of 600 met minutes/week), and a 95% power to detect an increase in prevalence of adequate consumption of fruit and vegetables (5 or more portions per day) from 30% to 50%.

### Recruitment

In the prior cross-sectional study the list of employees (n = 1743) was organised according to random numbers generated by computer [15]. Employees who were randomly included in the list generated were invited to participate in the study, initially by email and then with a follow up phone call if they did not respond.

### Intervention

The intervention took place from November 2015 to November 2017 using participatory action research with a co-operative inquiry group (CIG) and is more fully described in a separate publication [24]. The intervention focused on four key areas:

- *The provision of food*: The CIG negotiated with the catering contract manager to offer and promote a subsidised ‘wellness meal’ (a low fat, low salt option with additional vegetables and fruit) for all employees on all shifts. In addition, they sold affordable healthy snacks at the cafeterias and made free fruit available as snacks throughout all shifts. Healthier snacks, such as fruit, were provided during power outages when the power plant was taken out of production for maintenance and when staff worked longer hours in shifts.
- *Opportunities for physical activity*: Areas were identified within the surrounding nature reserve for walking, running and cycling. A ‘First Friday’ sports day, occurring once a month, was rostered on the organizational plan as a regular activity that encouraged employees to participate in physical activity. In addition, functional exercise classes were conducted four times per week during the intervention. Employees were also encouraged to attend weekly park run events that were organised in their local communities.
- *Provision of health and wellness services*: CIG members collaborated with the information technology and the communications department to educate employees on healthy living, using a dedicated wellness newsletter and live broadcasting on plasma screens throughout the workplace. Employees received two health risk assessments (HRA) during the intervention. Following the HRA they were given feedback on their risk profile. In addition, clinical staff were trained to offer brief behaviour change counselling [25] to those that wanted to discuss their risks further. Private health insurers also offered additional health assessments by tracking risk factors and health behaviours.
- *Leadership buy-in and participation*: Results of the employees’ HRA and the design of the WHPP were presented to the managerial team at the beginning of the intervention. The managers’ health profiles were also assessed. Managers approved the prevention programme and led by example in choosing wellness meals, marketing activities, participating in physical activities and promoting health by broadcasting discussions of their own behaviour change.

### Data collection

All the questionnaires and clinical tests were administered by trained health professionals from the Health and Wellness Department at testing booths in close proximity to employees’ workstations during both day and night shifts. Information was collected at baseline and at 24 months.

#### Questionnaire

A questionnaire collected data on demographic information, medical and family history, medication use, psychosocial stress, tobacco smoking, diet, physical activity and alcohol consumption.

Psychosocial stress was measured by asking respondents to select the biggest causes of stress at work or at home from a list of 12 items (see Table 2). Questions on tobacco smoking as well as fruit and vegetable intake were extracted from the South African Demographic Health Survey Questionnaire [26].

**Table 1.** Participation in healthy choices at work programme over two years (N = 137)

HCW activities	%
Ate wellness meal > 1/week	44.5
Often/always ate fruit provided during outage	62.0
Often/always participated in physical activity during outage	5.1
Often/always participated in monthly First Friday sport	27.7
Often/always attended weekly functional exercise session	2.2
Often/always participated in weekly lunchtime walks	32.8
Often/always participated in weekly lunchtime runs	7.3
Often/always participated in weekly lunchtime cycles	4.4
Ever attended community park run events (yes/no)	24.1
Participated in biggest loser competition (yes/no)	8.8
Obtained health risk assessment feedback (yes/no)	100.0
Received behaviour change counselling (yes/no)	80.3

**Table 2.** Change in psychosocial factors causing stress (N = 137)

Stress factors	Baseline %	Follow up %	p value
**Work**			
Relationship with colleagues	21.1	11.3	0.015
Lack of recognition	18.8	18.8	1.000
Lack of resources to do my work	29.5	30.3	1.000
Lack of meaningful work	15.1	13.5	0.664
Relationship with my supervisor	9.8	12.0	0.664
Lack of clarity concerning work outputs	20.9	15.7	0.265
**Personal**			
Personal finances	29.8	18.3	0.008
My health or family member’s health	22.3	10.8	0.006
Relationship with family/children	16.0	13.7	0.690
Relationship with my partner/spouse	13.3	9.2	0.210
Emotional/mental health concerns	8.8	4.7	0.227
I have challenges with addictions	3.3	0.8	0.250

The Global Physical Activity Questionnaire (GPAQ) was used to quantify levels of physical activity [27]. MET (metabolic equivalent of task) minutes were calculated from the GPAQ data. The MET is a physiological measure that expresses the energy cost (or calories) of different levels of physical activities [28]. A MET minute score was calculated by multiplying the MET value for the type of activity by the time in minutes that the activity was performed. A minimum of 600 MET minutes per week was required to be considered physically active. This translates to participating in a minimum of 150 minutes of moderate-intensity activity per week or 75 minutes of vigorous intensity activity per week.

Data on alcohol use was collected using the validated Alcohol Use Disorders Identification Test (AUDIT) questionnaire [29]. The AUDIT questionnaire included 10-questions with a 4-point Likert scale that gave a possible total score of 41. Respondents with a score of 7 or less were categorised as sensible drinkers, those with a score of 8–19 as potentially harmful drinkers and those with a score of 20 or more as potentially dependent drinkers.

At follow up, an additional questionnaire assessed participation in the various components of the HCWP (Table 1)

#### Clinical measurements

Systolic and diastolic blood pressure was recorded using a standardised operating procedure and automated sphygmomanometer (Microlife AG, 9943, Switzerland) [30]. Random blood glucose and total cholesterol testing was conducted with a capillary blood sample using a Cardio Chek (Polymer Technology Systems, USA) [31].

Standing height (cm) was measured to the closest 0.1 cm with a stadiometer. Body mass was recorded to the closest 0.1 kg using a portable calibrated scale (Seca 813, UK). Body mass index was then calculated and participants were classified as ideal weight, overweight or obese [32].

Waist and hip circumference were measured using a stretch resistant tape and standardised operating procedure and the waist to hip ratio (WHR) was calculated [33].

The 10-year risk of death from myocardial infarction or stroke was assessed using a validated non-laboratory algorithm for South Africa [34]. The estimated risk was categorised into low risk (< 10%), moderate risk (10–20%) and high risk (> 20%).

### Data analysis

Data were then analysed using the Statistical Package for the Social Sciences Version 24.1.

Descriptive analysis was used to calculate the mean and standard deviation or frequency and percentage of all variables at baseline and follow up. Paired t-tests were used to compare the mean differences for normally distributed numerical data (e.g. blood pressure, blood glucose, total cholesterol, BMI, waist circumference, WHR) from baseline to follow up at two years. McNemar’s Chi-square test was used to compare paired binary categorical data from before to after (e.g. psychosocial stress, smoking, fruit and vegetables).

## Results

**Figure 1.**
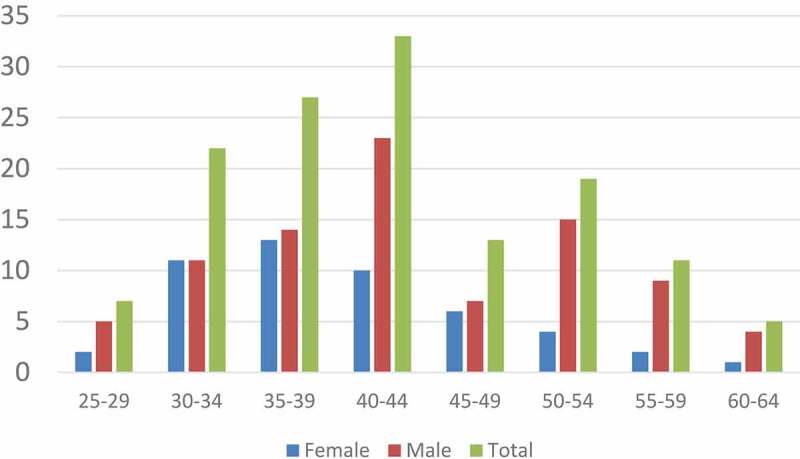
Age and sex distribution of participants (N = 137)

Paired data was obtained for 137 participants (73% of sample) who completed all the questionnaires and assessments. The mean age of the participants was 42.7 years (SD 9.7), 64% were male and the distribution is shown in Figure 1. Overall, 4% were senior managers, 13% were middle managers, 58% were supervisors or professionals and 24% were general employees.

### Participation in wellness activities

Table 1 shows the proportion of participants that engaged in the various HCWP activities. All the employees received feedback on their HRA and the majority received behaviour change counselling and consumed healthier snacks during outages. A substantial proportion purchased the wellness meals daily and participated in the monthly sport events, daily lunchtime walks or park runs. Very few participated in physical activity during power outages, functional exercise classes, lunchtime runs or cycles and the competition to lose weight.

### Change in psychosocial stress factors

Table 2 presents results for self-reported stressors at work and home for all participants. Participants reported significantly less stress (p < 0.05) from personal finances, relationships with colleagues as well as from their own health or that of a family member at follow up.

### Behavioural risk factors

Table 3 reports on the changes in behavioural risk factors. Significantly fewer employees engaged in harmful alcohol consumption at the follow-up assessment. Other lifestyle behaviours that improved significantly were fruit and vegetable consumption and levels of physical activity. There was no significant reduction in smoking.

**Table 3.** Change in behavioural risk factors (N = 137)

	Baseline %(95% CI)	Follow up % (95% CI)	p value
Sensible alcohol drinker (AUDIT score < 8)	78.2 (70.4: 84.8)	93.5 (90.5: 98.6)	0.001
Harmful alcohol drinker (AUDIT score 8–19)	21.0 (14.5: 28.8)	4.8 (2.0: 9.7)	
Dependent alcohol drinker (AUDIT score ≥ 20)	0.8 (0.1: 3.7)	1.6 (0.3: 5.1)	
Tobacco smoking	25.0 (18.0: 33.1)	21.8 (15.2: 29.6)	0.344
Inadequate fruit & vegetable intake (< 5 portions/day)	73.2 (64.9: 80.4)	35.8 (27.7: 44.5)	˂0.001
Insufficiently active (< 600 MET minutes/week)	55.9 (46.9: 64.7)	34.7 (26.6: 43.6)	˂0.001

### Metabolic risk factors

Table 4 shows the changes in metabolic risk factors. There was a significant improvement in systolic and diastolic blood pressure. There was also a significant decrease in cholesterol levels, but not in random blood glucose. Weight, BMI and waist circumference were unchanged.

**Table 4.** Change in metabolic risk factors (N = 137)

Risk factors	Baseline Mean (SD)	Follow up Mean (SD)	Mean of the difference (95% CI)	p value
Systolic blood pressure (mmHg)	131.6 (18.5)	121.4 (14.6)	−10.2 (−7.3: −13.2)	< 0.001
Diastolic blood pressure (mmHg)	83.4 (13.7)	79.5 (8.8)	−3.87 (−1.8: −5.8)	˂ 0.001
Total cholesterol (mmol/l)	5.6 (1.1)	5.1 (1.1)	−0.45 (−0.3: −0.6)	˂ 0.001
Random glucose (mmol/l)	5.7 (1.5)	6.0 (2.0)	0.31 (−0.6: 0.2)	0.069
Body mass index (kg/m^2^)	29.0 (5.5)	29.0 (5.7)	−0.05 (−0.4: 0.3)	0.760
Waist circumference (cm)	92.1 (14.3)	92.2 (14.4)	0.05 (−1.1: 1.0)	0.926
Waist to hip ratio (cm)	0.86 (0.1)	0.87 (0.1)	−0.00 (−0.0: 0.0)	0.484

### Non-communicable diseases

Prevalence of self-reported NCDs and the use of medication indicated that changes were not significant from before to after (Table 5).

**Table 5.** Prevalence of self-reported non-communicable disorders and medication use

	Diagnosis of NCD			Medication for NCD		
	Baseline % (95% CI)	Follow up % (95% CI)	p value	Baseline % (95% CI)	Follow up % (95% CI)	p value
Diabetes	6.0 (2.9: 10.9)	9.0 (3.4: 11.9)	0.317	7.0 (3.5: 12.4)	8.6 (4.7: 14.4)	0.625
Hypertension	15.9 (10.4: 22.7)	18.9 (10.4: 22.7)	0.311	14.0 (8.8:20.7)	18.6 (12.8: 26.0)	0.146
High cholesterol	16.4 (10.9: 23.4)	14.9 (11.5: 24.2)	0.303	13.0 (8.0: 19.5)	13.7 (8.7: 20.4)	1.000
Heart condition	6.0 (2.9: 10.9)	4.5 (2.9: 10.9)	0.687	4.5 (1.9: 9.1)	3.8 (1.4: 8.0)	1.000
Lung condition	5.3 (2.4: 10.0)	6.1 (2.4: 10.0)	1.000	3.7 (1.4: 8.0)	3.0 (1.0: 6.9)	1.000
Cancer	2.3 (0.6: 5.9)	3.0 (1.0: 6.9)	1.000	1.5 (0.36: 4.7)	0.7 (0.1: 3.4)	1.000
Depression	15.0 (9.7: 21.8)	13.5 (9.7: 21.8)	0.250	11.2(6.7: 17.3)	10.4 (6.1: 16.5)	1.000

### Cardiovascular risk score

Table 6 presents the 10-year cardiovascular disease risk score for participants. There were no significant risk reductions. However, there was a 4.5% absolute risk reduction for employees who were categorised as high risk, suggesting some improvement.

**Table 6.** 10-year cardiovascular disease risk (N = 137)

Variable	Baseline % (95% CI)	Follow Up % (95% CI)	p value
Low risk	66.2 (57.8: 73.8)	67.7 (59.4: 75.2)	0.155
Moderate risk	18.0 (11.2: 44.5)	21.1 (7.2: 34.8)	
High risk	15.8 (6.8: 39.2)	11.3 (6.0: 44.4)	

## Discussion

The HCWP was associated with significant improvements in certain risk factors for NCDs. Significant changes in behavioural risk factors included a reduction in harmful alcohol use, increase in fruit and vegetable intake and improvement in levels of physical activity. Employees’ systolic and diastolic blood pressure, as well as serum cholesterol concentration, improved significantly. Employees reported less stress from relationships at work and from their own health. There was no change in tobacco smoking and the prevalence of overweight and obesity. There was a non-significant reduction in the proportion of employees at high cardiovascular risk. The proportion of employees diagnosed with NCDs and on medication did not change.

Despite a policy of zero alcohol tolerance in the workplace, with routine breathalyser testing, there was a high prevalence of harmful alcohol use, which was similar to other workplace settings [35,36], and which reduced significantly. The AUDIT was a useful tool to identify excessive drinking amongst employees as well as for the research. Such screening and feedback enables employees who are not dependent on alcohol to stop or reduce their alcohol consumption [37]. The study contributes to the evidence on effectiveness of AUDIT as part of interventions to reduce harmful alcohol use [38]. Brief behaviour change interventions are also effective [39] and it is likely that this counselling was the component most responsible for the reduction in harmful alcohol use.

Fruit and vegetable intake improved significantly with 64% of participants consuming the recommended daily portions. It is likely that the combination of easy access to healthier food, education and cooking demonstrations, were responsible for the change in diet [40–42]. The improved fruit and vegetable intake should be clinically meaningful and reduce the risk of NCDs such as type 2 diabetes [43].

The baseline prevalence of insufficiently active employees (56%) was higher than the South African population [43], although lower than figures reported for other South African workplaces [44]. The beneficial effect of this HCWP may partly be due to the longer duration of the intervention (24 months) as studies with shorter interventions may be less effective [35]. A systematic review on workplace-based interventions for improving physical activity also supported the need to target change in the environmental aspects of the organisation [36]. The opportunities for physical activity during working hours may have been critical as behaviour change counselling alone may not be sufficient in the workplace [39].

A quarter of the workforce smoked tobacco and there was no significant reduction during the study. The non-significant reduction of 3.2% is similar to that seen in workplaces that become totally smoke free [45]. The power plant was not a completely smoke free workplace and provided many facilities where people could smoke. The brief behaviour change counselling and feedback on individual risk profiles possibly accounted for the reduction. Many participants indicated a willingness to stop smoking, but needed help. Smoking cessation programs were only available through private health insurers, which were not integrated into the HCWP.

The change in blood pressure is likely to be clinically significant as a 10 mmHg reduction in systolic blood pressure translates to a 22% reduction in coronary heart disease and 41% reduction in stroke [46,47]. The workplace may be particularly suited to interventions that reduce blood pressure as even much shorter interventions can have a significant, albeit smaller, effect [35]. The change in blood pressure does not appear to be due to an intensification of treatment as this was unchanged during the intervention. It is possible that reductions in blood pressure were related to increased levels of physical activity, reduced alcohol intake and changes in diet as well as greater awareness of the need to control hypertension with feedback from the HRA and behaviour change counselling [40–42]. Behaviour change counselling has a 55% increased chance of instilling positive outcomes compared to routine medical advice in reducing blood pressure, cholesterol levels, BMI, harmful drinking and smoking [48,49].

The change in total cholesterol was also likely to be clinically significant as a decrease of 0.6 mmol/l is associated with a 50% reduced risk for ischaemic heart disease at age 40 [50]. The reduction in cholesterol may be attributed to increased physical activity and possibly changes in diet [42].

The prevalence of overweight and obesity in our participants was similar to those from other South African and international workplaces [45,51–53]. BMI did not improve despite the improvements in physical activity and fruit and vegetable intake. This may be because other aspects of the diet did not improve, such as the consumption of carbohydrates, fat and oil, which were not measured. Although healthier food was more accessible and incentivised at work, the intervention did not directly target the diet at home or outside of work. Other family members and eating habits may have continued to influence what was cooked and eaten outside of work and negated the effect of the HCWP on weight loss.

The reduction in stress from personal finance can be linked to weekly financial wellness workshops and one-on-one consultations conducted in the organisation, which were not part of the HCWP. The improvement in relationships with colleagues could be associated with the various sports activities, which also facilitated team building. Improved perceptions of personal health are congruent with the HCWP and its effects on physical activity, diet, alcohol and metabolic risk factors. Other psychosocial risk factors did not change and this is also congruent with the loss of skilled employees abroad and an organisational culture characterised by cost-cutting and financial restraints during the same period as the study.

This study met the two criteria for offering comprehensive and effective WHPPs for NCDs as defined by Healthy People 2010: health education, which includes a behaviour change component, as well as a supportive social and physical environment. Only 7% of WHPPs with large organisations in high income countries met these criteria [54]. The design of the intervention itself through participatory action research was an important factor in the success of our HCWP and this was evaluated in a previous study [13].

### Limitations

A control group was not included in the study as the intervention targeted the whole organisation and therefore all employees were potentially affected by the HCWP. It would have been difficult to enrol a similar workforce from another company to act as a control. Prior to the HCWP, annual HRAs showed a steady increase in cardiovascular risk profile. Indeed, the study was prompted by eight deaths from NCDs within the company in 2014. The reductions in risk factors for NCDs associated with the HCWP are therefore unlikely to be due to trends in the study population that were not controlled for. In addition, there were no other known interventions targeting the workforce at the power plant during this period. Nevertheless, the reductions in risk factors should be seen as associated with the HCWP and the study cannot prove cause-and-effect.

The study sample size was too small to conduct further sub-group analysis and the study was only powered to measure differences in the key outcomes from before to after the intervention and not to measure relationships between variables.

It was necessary to invite 395 people to obtain the sample size of 156 as many people did not respond to the invitation via email or telephone, and this was most likely due to them being unaware of the invitation rather than deciding not to participate. We believe that many of the non-responders were away on training in partner commercial plants outside of South Africa, working in other regions, working shifts, pregnant or on extended sick leave. Only four employees that were invited declined to participate. Although we had limited data on the non-responders, we were able to show that they did not differ significantly from those that did respond in terms of their role within the company.

Reasons for loss to follow up (n = 19) included employee resignations (to a new recruiting commercial power plant), retirements, relocation (to other business units) and five withdrawals from the study. However, there were no significant differences between those that were lost to follow up and those that remained in the study in terms of key baseline characteristics.

### Implications

This study adds to the body of evidence supporting the value of WHPPs as a means to prevent NCDs. As discussed, if these reductions in risk factors were sustained then they should lead to a reduced incidence of myocardial infarction, stroke and type 2 diabetes. If the HCWP is sustained then it should impact new employees from an earlier age, as they join the company, and have even greater benefits in terms of preventing NCDs. The sample in this study were mostly men and under the age of 45-years. There is therefore potential for prevention of NCDs, which mostly present over the age of 45 years, particularly for ischaemic heart disease, which is more common in men [55,56]. This study, therefore, suggests that more attention should be given to the workplace as a setting for effective disease prevention in order to meet national and international [35] targets for the prevention of NCDs.

It would be valuable to follow up the same workforce overtime to evaluate the sustainability and longer term effects of the HCWP. Since the study ended a state-of-the-art exercise and fitness facility has been completed. It would be beneficial to strengthen the smoking cessation program and to remove the ‘smoking shelters’ that encourage this behaviour on site. A broader approach to healthy eating at home and the workplace, as well as broader evaluation of eating habits may be needed to enable weight loss. Improvements will be encouraged if the HCWP is more strongly linked to employees’ benefits. It would also be useful to replicate and evaluate the intervention in other similar workplace settings where it might be possible to also include a control group in a stronger experimental study design.

## Conclusion

This HCWP was associated with clinically significant improvements in behavioural, metabolic and psychosocial risk factors for NCDs. The study shows the potential of health promotion in the workplace to complement interventions in the health services and community. Workplace-based interventions could make a substantial contribution to preventing the burden of disease from NCDs in South Africa. Our findings included reductions in alcohol use, improvements in fruit and vegetable intake, increase in physical activity levels, as well as improving metabolic risk factors for blood pressure and cholesterol over two years. This study contributes to research from low- and middle-income countries on the effectiveness of WHPPs for reducing risk factors for NCDs.

## Paper context

Workplace health promotion programmes to reduce risk factors for NCDs, predominantly rely on single component interventions and are evaluated in high-income settings. This paper reports how a multi-component Healthy Choices at Work Programme, was associated with significant improvements in risk factors for NCDs. Key features of the programme included leadership from a diverse team, a focus on personal behaviour change with health risk assessments, feedback and counselling as well as social engineering of the environment to make the healthy choice, the easy choice.
